# Investigating the Ultrasonication‐Induced Changes in the Physicochemical, Phytochemical, Microbial, and Sensory Properties of Cantaloupe–Sugarcane Blend Juice

**DOI:** 10.1002/fsn3.70519

**Published:** 2025-06-27

**Authors:** Abdul Rehman, Muhammad Nadeem, Mian Anjum Murtaza, Safdar Hussain, Nida Firdous, Rizwan Arshad, Ashiq Hussain, Abdeen Elsiddig Elkhedir

**Affiliations:** ^1^ Institute of Food Science and Nutrition University of Sargodha Sargodha Pakistan; ^2^ Department of Physics University of Sargodha Sargodha Pakistan; ^3^ Department of Food Science and Technology MNS‐University of Agriculture Multan Multan Pakistan; ^4^ Department of Allied Health Sciences The University of Chenab Gujrat Pakistan; ^5^ Sudan University of Science & Technology Khartoum Sudan

**Keywords:** bioactive compounds, green bioprocess technology; storage analysis, juices preservation, ultrasonication

## Abstract

Sonication is an emerging technology that is being rigorously researched for being implemented across several food commodities. The impact of sonication (70% amplitude, 25 KHz frequency, and 525 W power) durations (T1 [2 min], T2 [4 min], T3 [6 min], T4 [8 min], and T5 [10 min]) and a chemical preservation treatment named CSJ+ on cantaloupe–sugarcane blend juice was investigated in this study, keeping CSJ0 as a negative control. The effect of different sonication times and chemical preservation, along with the control, on 120‐day storage at refrigerated temperature was also assessed during regular intervals. The results showed that sonication positively influenced the nutritional quality of the juice blend by significantly (*p* < 0.05) enhancing the total phenolic, flavonoid, and antioxidant characteristics. The pH values of all treatments decreased, whereas titratable acidity significantly increased after storage. A notable reduction in brix and an increase in viscosity occurred with the extension of the storage time. After completion of the storage period, maximum vitamin C was noted in T3 (37.168 mL/100 mL) and minimum in T5 (16.66 mL/100 mL). The highest total phenolic content was noted in T5 (170.8 mg GAE/100 mL) at the start of storage after ultrasonication, while maximum retention was observed in T3 (154.6 mg GAE/100 mL) at the end of storage. Similarly, an increasing trend in total flavonoid content and antioxidant activity after ultrasonication was also recorded. The total plate count and yeast and mold count rose over prolonged storage across all treatments. Sensory evaluation also showed significantly (*p* < 0.05) different results for all treatments and storage days, while T3 was preferred by the evaluators. Thus, sonication with an optimized time (6 min) period may be effectively utilized to enhance the nutritional and sensory qualities of the sugarcane‐cantaloupe blend juice from the customers' perspective.

## Graphical abstract

Sonication at optimum operating conditions is an advancing green processing technology for processing and preservation of fruit juices. This treatment enhanced the juice's phytochemical contents and antioxidant activity, suggesting its potential health advantages. Cantaloupe–sugarcane blend juice sonicated for 6 min has shown higher nutritional, sensory, and phytochemical properties.

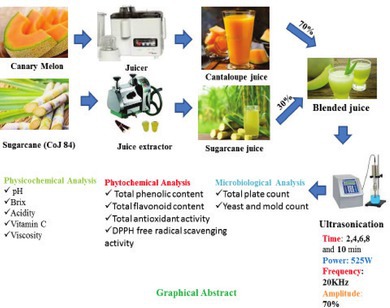

## 1 Introduction

Fruits and vegetables, especially melons, are rich in antioxidant polyphenols, carotenoids, minerals, and vitamins, and these bioactive substances contribute to illness prevention (Yıkmış 2020; Yadav et al. 2025). They also supply dietary fiber, which is linked to a decreased risk of cardiovascular disease and obesity (Hussain, Laaraj, Tikent, et al. 2024; Pérez‐Juárez et al. 2024). Melons are considered a rich source of micro and macro nutrients, including potassium and magnesium, as well as vitamins A and C (Fundo, Miller, Garcia, et al. 2018). These are also considered an excellent source of different amino acids such as citrulline, glutamine, and γ‐aminobutyric acid (Singh et al. 2020). Cantaloupe ( *Cucumis melo* L.) belongs to the Cucurbitaceae family, and this fruit is widely used in both cooking and medicine. It is mostly grown and eaten in Europe and is one of the several economically significant species in the Cucurbitaceae family (Vella et al. 2019). In 2020, 28.5 million tons of melons were produced globally. China leads the globe in melon production with 13.8 million tons, followed by Turkey with 1.7 million tons (Adıgüzel et al. 2023). Cantaloupe is a popular melon variety known for its appealing taste and flavor, loads of bioactive compounds, appreciable nutritional contents, and several health benefits. This fruit has a lot of uses in the food industry for the development of a wide range of food products (Jorge et al. 2022). Thus, a cantaloupe juice combination with some other juice and then the application of green processing technology could provide consumers a new formulation with ensured quality and safety.

Sugarcane ( *Saccharum officinarum* L.) is a perennial grass of the Poaceae family, cultivated in more than 90 countries worldwide for its economic value and high productivity. It demonstrates antioxidant characteristics attributed to phenolic substances, including phenolic acids and flavonoids (Fatima et al. 2023). An 8‐oz serving of sugarcane juice has 250 cal and 30 g of natural sugars, and this fresh juice is normally free from additives. It is devoid of fat, cholesterol, and protein, while containing salt, potassium, calcium, magnesium, iron, and fiber (Mukhtar et al. 2022). It consists of water (75%–85%), non‐reducing sugars (10%–21%), reducing sugars (0.3%–3%), organic compounds (0.5%–1%), inorganic compounds (0.2%–0.6%), and nitrogenous compounds (0.5%–1%) (Sreedevi et al. 2018). Thermal treatment can diminish microbial count and enzymatic browning in sugarcane juice. Nonetheless, it may also induce the Maillard reaction process and expedite the decomposition of chlorophyll and phenolic components, hence altering sensory and nutritional attributes of the juice (Huang et al. 2015). Therefore, the use of non‐thermal processing technologies could be beneficial in avoiding such reactions. Ultrasound is a rapidly advancing non‐thermal technique that enhances quality and ensures safety, especially in food containing heat‐sensitive nutritional, sensory, and functional characteristics (Siddique et al. 2023; Zhang et al. 2024). This preservation technique has been more popular in the last decade. It has been utilized in the fruit juice and beverage industry due to its numerous advantageous qualities (Hussain, Batool, Sidrah, et al. 2024). The process is simple, economical, reliable, environmentally benign, and highly effective in preserving juices with enhanced quality attributes (Dolas et al. 2019; Hussain, Batool, Yaqub, et al. 2024). The amalgamation of cantaloupe–sugarcane juice was executed to develop a distinctive beverage that integrates the nutritional advantages of both components. Cantaloupe, abundant in vitamins A and C, potassium, and antioxidants, was combined with sugarcane juice, which is a significant source of sucrose, minerals, and phytochemicals. The blending procedure may result in enzymatic browning, oxidation, and microbial proliferation, thus undermining the quality and safety of the juice. Traditionally, thermal methods like pasteurization and sterilization have been utilized to conserve the fruit juices. Nevertheless, these methods may result in the heat destruction of nutrients, taste, and color, thus undermining the overall quality of the juice. Conversely, ultrasonication provides a non‐thermal preservation method that can deactivate enzymes, decrease microbial burden, and enhance the stability of the juice while preserving its nutritional and sensory attributes. Ultrasonication emphasizes the preservation of bioactive components, taste, and color, rendering it a superior procedure compared to traditional heat treatments. Thus, this research was carried out to assess the impact of non‐thermal treatments (chemical preservation and sonication) on the physicochemical, phytochemical, and microbial properties of cantaloupe–sugarcane juice blends during storage. To examine the appropriateness of sonication duration on cantaloupe–sugarcane blended juice using various analyses.

## 2 Material and Methods

### 2.1 Purchasing of Raw Materials

Cantaloupe ( *Cucumis melo* L.) with an average weight of 2 kg/fruit (*n* = 25) was acquired from the local fruit market in Sargodha, Pakistan. Fresh samples of sugarcane (variety Tritan 44), having uniform color, shape, and size (*n* = 50) were sourced directly from the fields of Sargodha, Pakistan. The flora utilized in this study was gathered in compliance with regional or federal regulations of the country. The cantaloupe–sugarcane utilized in this investigation were conveyed to the laboratory of the Institute of Food Science and Nutrition at the University of Sargodha, Sargodha, where after botanical identification was done before further processing.

### 2.2 Chemicals and Reagents

All chemicals employed in this study were of analytical grade, obtained from Sigma Aldrich (Seelze, Germany), and were available in the local market of Sargodha, Pakistan. Identical trade/brand chemicals were utilized for each analysis to avoid any variation of the results. Distilled water was used for each analysis.

### 2.3 Preparation of Juice Blends

#### 2.3.1 Preparatory Operations of Raw Material

Juice was extracted from healthy, uniformly sized sugarcane that was free of disease and attached green plant components. The sugarcane was properly cleaned and washed with running tap water before being used. Fully grown and healthy cantaloupe was smashed and cleaned with tap water to extract the juice for use in the juice blend. This process was carried out under the standard laboratory conditions using an aseptic environment.

#### 2.3.2 Extraction of Juices for Blend

First of all, washing of cantaloupe and sugarcane was done. The peel and seeds of the cantaloupe fruit were removed to extract juice using a laboratory blender (P105‐Panasonic Japan). A helical presser was used to extract juice from the crushed sugarcane, and samples were kept chilled in sealed plastic bottles. On the basis of sensory evaluation, both juices (30 mL sugarcane juice and 70 mL cantaloupe juice) were thoroughly mixed to form a blend. The blended juice was filtered through a sieve with 0.8 mm pores to get rid of any unnecessary impurities that may be present in the juice. All of the juice preparation and preservation processes were carried out using the methodology presented by Khandpur and Gogate (2015), with the appropriate modifications. The treatment plan followed during the study is mentioned below in Table 1.

**TABLE 1:** Treatment plan for cantaloupe–sugarcane blended juice.

Treatment	Use of preservatives/Ultrasonication	Frequency/Concentrations	Power (W)	Amplitude (%)
CSJ_+_	Positive control	C_7_H_5_NaO_2_, K_2_S_2_O_5_, and C_6_H_7_KO_2_ @1 g/L	—	—
CSJ0	Negative control	—	—	—
T1	2 min ultrasound	Ultrasonication 20 KHz	Ultrasonication 525 W	70%
T2	4 min ultrasound	Ultrasonication 20 KHz	Ultrasonication 525 W	70%
T3	6 min ultrasound	Ultrasonication 20 KHz	Ultrasonication 525 W	70%
T4	8 min ultrasound	Ultrasonication 20 KHz	Ultrasonication 525 W	70%
T5	10 min ultrasound	Ultrasonication 20 KHz	Ultrasonication 525 W	70%

Abbreviations: CSJ+, Positive control; CSJ0, Negative control; T1, 2 min ultrasound; T2, 4 min ultrasound; T3, 6 min ultrasound; T4, 8 min ultrasound; T5, 10 min ultrasound.

#### 2.3.3 Chemical Preservation

The blended juice was evaluated against the control and other chemical treatments (sodium benzoate, potassium metabisulphite and potassium sorbate) @ 1 g/L that were included in the treatment (CSJ+). The treatment protocol is defined in Table 1.

#### 2.3.4 Ultrasonication of Juice

The blend juice of cantaloupe–sugarcane underwent an ultra‐sonication process according to the methodology employed by (Hussain, Batool, Sidrah, et al. 2024), with some modifications. The ultrasonic processor (UP400S, Hielscher Ultrasonics GmbH, Hielscher USA Inc.) equipped with a 0.5‐in. probe was utilized for sonication. The juice underwent ultrasonic treatment (5 s on and 5 s off, 70% amplitude, and 20 kHz frequency) for several durations (2, 4, 6, 8, and 10 min) at 20°C (Table 1). Sonication of the blended juice was conducted immediately following the extraction and acquisition of cantaloupe–sugarcane juice. The analysis was conducted under darkness. The non‐sonicated and untreated fresh sample was designated as the control.

#### 2.3.5 Storage of Juices

Untreated, chemically preserved, and sonicated blended juice samples were stored in airtight plastic bottles at 4°C for 120 days post‐processing analysis.

### 2.4 Physicochemical and Phytochemical Analyses

Sample placed in refrigerator after preservation techniques were used for physicochemical analysis including pH, brix, acidity, and vitamin C, and phytochemicals analysis including total phenolic content, flavonoid content, and antioxidant activity, and lastly microbiological analysis, which included total plate count and mold count. The sensory evaluation was also performed to study the effect of chemicals and ultrasonication on cantaloupe–sugarcane blended juice.

#### 2.4.1 Determination of Total Soluble Solids (TSS)

Total soluble solids (TSS) of the blended juice were quantified utilizing a computerized refractometer equipped with automated temperature correction. The digital hand refractometer (Ebro Electronic GmbH, Ingolstadt, Germany, Model: DR‐10) has measurement range ranging from 0°B to 54°B (Kassebi et al. 2022).

#### 2.4.2 Determination of pH

According to the method provided by Rydzak et al. (2020), with slight modifications a digital CP‐411 pH‐meter (Elmetron, Zabrze, Poland) was used to measure the pH of cantaloupe–sugarcane blended juice at room temperature (20°C ± 1°C). An electrode of pH meter was put into 10 milliliters of blended juice, and the pH was measured after stabilizing the readings. Three triplicates of sample were recorded for more exact and reliable findings.

#### 2.4.3 Determination of Titratable Acidity

The titratable acidity of blended cantaloupe–sugarcane juice was determined using the standard procedure established by Talasila et al. (2012), with slight changes. Two grams of concentrated fruit juice were diluted with 9 mL of distilled water and subsequently filtered using Whatman No. 1 filter paper. Two drops of a 1% phenolphthalein indicator were introduced to 10 mL of the filtrate and titrated with 0.1 N NaOH (4 g NaOH in 1000 mL of distilled water) until a pink endpoint was achieved. The total titratable acidity was determined using the equation shown below.

$$\mathrm{TTA}\;\left(\%\right)=\frac{\left(0.1\text{NNaOH}\times 0.064\times 100\right)}{V}$$

#### 2.4.4 Determination of Vitamin C

The procedure provided by (Hussain, Kausar, Siddique, et al. 2024), with slight modifications, was used to assess the amount of vitamin C in sugarcane and cantaloupe blended juice. The standard reference was 0.1% ascorbic acid, and the detection dye, 2,6‐dichlorophenolindophenol (DCPIP), was utilized (0.1 g of dye was dissolved in distilled water). After adding 3 mL of DCPIP dye to a test tube, the solution was titrated with either SCJ sample or 0.1% ascorbic acid until it turned clear. The amount of vitamin C contained was determined by measuring the volume of blended juice used to decolorize the DCPIP dye; the findings were expressed as mg of ascorbic acid per 100 mL of juice.

#### 2.4.5 Determination of Total Phenolic Content

The total phenolic content was determined using a modified Folin–Ciocalteu reagent method as outlined by Hussain et al. (2021), with some modifications. One milliliter aliquot of diluted blended juice was mixed with 1 milliliter of the 10% Folin–Ciocalteu reagent. Subsequent to vortexing, 2 mL of a 20% sodium carbonate solution was added to the mixture. After 60 min incubation at 30°C in darkness, the absorbance was measured at 760 nm using a spectrophotometer (Halo DB‐20, UV–VIS double beam). The results were quantified in milligrams of gallic acid equivalent (GAE) per 100 mL of juice.

#### 2.4.6 Determination of Total Flavonoid Content

The total flavonoid content of the blended juice was determined using the approach of Hussain et al. (2021) with some modifications. During this procedure, a 1.5 mL aliquot of diluted blended juice was mixed with 75 μL of a 5% sodium nitrite solution. After 1 min of vortexing, 150 μL of a 10% aluminum chloride solution was added. Subsequently, 0.5 mL of 1 M NaOH was introduced, and absorbance was measured at 510 nm using a spectrophotometer (Halo DB‐20, UV–VIS double beam, Diepoldsau, Switzerland). The results were calculated in micrograms of catechin equivalent (CE) per 100 mL of juice.

#### 2.4.7 Determination of DPPH Free Radical Scavenging Activity

The capacity of juice to scavenge free radicals was assessed utilizing the methods outlined by (Jabbar, Abid, Hu, Muhammad Hashim, et al. 2014), with slight modifications. Briefly explaining, 1 mL of juice extract was combined with 2 mL of DPPH solution (60 μmol in ethanol). Absorbance was measured at 517 nm using a spectrophotometer after a 30 min incubation time in the dark. The identical process was employed to produce the control, which is ethanol. The subsequent method was employed to calculate the percentage inhibition radical scavenging activity or RSA (%) predicated on the reduction in absorbance of juice samples:

$$\mathrm{RSA}\;\left(\%\right)=\frac{A\;\left(\text{control}\right)-A\;\left(\text{sample}\right)}{A\;\left(\text{control}\right)}\times 100$$

Where A (sample) represents the sample's absorbance and A (control) represents the control's absorbance. As a blank, ethanol was utilized. By using the previously stated test, the DPPH radical scavenging activity was also expressed as a μmol Trolox equivalent (TE)/mL juice. The tests were conducted in triplicates.

#### 2.4.8 Determination of Total Antioxidant Activity

The total antioxidant activity of the blended juice was evaluated using the method established by Navida et al. (2022), with some changing. Briefly, 1 mL of diluted cantaloupe–sugarcane blended juice was mixed with 4 mL of a reagent solution comprising 0.6 M sulfuric acid, 28 M sodium phosphate, and 4 M ammonium molybdate. After incubating the mixture for 95 min at 90°C, the absorbance was measured at 695 nm using a spectrophotometer. The results were calculated in milligrams of ascorbic acid equivalent per 100 mL of juice.

### 2.5 Microbial Analysis

#### 2.5.1 Total Plate Count

The blended juice was refrigerated (4°C ± 1°C) for 4 months after applying the selected preservation techniques (Table 1). Juice samples were examined for total plate count to assess the shelf stability of the juice samples after regular intervals (0, 30, 60, 90, and 120 days), as earlier described by Siddique et al. (2023). First of all, 1 mL sample of cantaloupe–sugarcane blended juice was transferred into a test tube using a sterile pipette, followed by the addition of 9 mL of saline solution for dilution. A decimal dilution of the sample was created by transferring 1 mL of the preceding dilution into 9 mL of saline solution. Fifteen milliliters of media, pre‐cooled to 45°C, were placed into plates within a 15 min interval, allowing sufficient time for solidification. Dilutions were adjusted in control plates and subsequently combined with sample dilutions and agar medium. Petri plates were incubated in an incubator at 35°C for 48 h. All colonies were enumerated, ranging from 30 to 300, and subsequently multiplied by the dilution factor. The results of total plate count were expressed as log_10_ CFU/mL of juice.

#### 2.5.2 Yeast and Mold Count

The yeast and mold count of cantaloupe–sugarcane blended juice was conducted according to the standard procedure used by Siddique et al. (2023). One milliliter of juice was collected in a test tube, and a turbid solution was produced with the addition of 9 mL of saline solution and subsequent agitation for 1–2 min. Subsequently, 1 mL of the 10 mL dilution was incorporated into the previously created 9 mL dilution. All dilutions were meticulously mixed. Thereafter, 12–15 mL of Potato Dextrose Agar (PDA) was introduced into a petri dish, the diluted sample was included, and the combination was allowed to solidify. All Petri plates were incubated at temperatures ranging from 20°C to 25°C. Following 48 h of incubation, the yeast and mold count in each plate was quantified and expressed as log_10_ CFU/mL of juice. Each analysis was performed in duplicate.

### 2.6 Sensory Evaluation

The cantaloupe–sugarcane blended juice evaluated by semi expert panel of 60 people, males and females, having 20–55 years of age, by following the procedure adopted from Rafique et al. (2023), using 9‐point hedonic rating scale. The availability and desire for the sugarcane and cantaloupe blended juice, was taken into consideration during selection of the panelists. For evaluation of juice, 30 mL samples that had been refrigerated (15°C), was presented in 100 mL transparent glasses and covered with plastic petri plates. Evaluations carried out in white light and at room temperature (25°C). The cantaloupe–sugarcane blended juice was rated on nine‐point hedonic scale of 1–9 (dislike very much to like very much) for color, flavor, taste and overall acceptability.

### 2.7 Statistical Analysis

Each assay was conducted in triplicate to derive the mean values. The statistical analysis of the collected data was conducted using Minitab 16 software, following the procedure established by Steel and Torrie (1981), employing the one‐way ANOVA test to identify significant differences in mean values at a significance level of *p* < 0.05. Tukey's test was employed to identify the significant differences among the values.

## 3 Results and Discussion

### 3.1 Ultrasonication Effect on pH of Cantaloupe–Sugarcane Blend Juice

The Table 2 shows that sonication treatment significantly impacted (*p* < 0.05) the pH of blended juice. pH is an essential attribute that influences the quality and shelf‐life of fruit and vegetable juices. The Table 2 illustrates the impact of the sonication procedure on pH over different intervals throughout a 120‐day storage period. Samples treated with sonication were compared with the positive control CSJ+ and the negative control CSJ0. At 0 days, the pH following sonication was elevated relative to non‐sonicated samples, with the highest pH seen in juice samples treated for 2 min. In the initial 30 days of storage, the pH in all treatments exhibited a small increase followed by a fall that continued until 120 days of storage. At the conclusion of the storage period, the lowest pH was recorded in treatment T5, where the sonication duration was set to 5 min. It may be inferred that pH lowers with storage. The decrease in pH value was more pronounced with additional sonication treatment. The reduction in pH of blended juice may result from several factors; sonication can lead to the disintegration of cellular components, releasing acidic substances such as organic acids, phenolic acids, and ascorbic acid (Zhang et al. 2024). Sonication may also stimulate enzymes like polyphenol oxidase (PPO), which can facilitate the oxidation of phenolic substances. This reaction may result in the creation of acidic chemicals and a reduction in pH (Taha et al. 2024). The results obtained align with Tiwari et al. (2009), who discovered that, the pH of orange juice rise immediately following sonication treatment. (Jabbar, Abid, Hu, Wu, et al. 2014) investigated the synergistic effects of sonication and acidification on certain qualitative attributes of carrot juice during storage. The findings of this study align with those of the current research, indicating a reduction in pH levels in the juices following sonication throughout the storage period.

**TABLE 2:** The mean values±SD for the effects of treatments on the physicochemical properties of blend (cantaloupe–sugarcane) juice during storage.

Treatments	Days	pH	Brix	Titratable acidity	Vitamin C (mL/100 mL)	Viscosity (mPa. s)
CSJ+	0	5.08 ± 0.057^mn^	12.07 ± 0.02^f‐i^	0.30 ± 0.010^c^	34.50 ± 0.500^kl^	0.08 ± 0.001^t^
CSJ+	30	5.05 ± 0.010^kn^	12.06 ± 0.06^f‐i^	0.31 ± 0.005^mo^	33.33 ± 0.152^m^	0.09 ± 0.001^rs^
CSJ+	60	5.00 ± 0.012^np^	11.99 ± 0.03^g‐i^	0.34 ± 0.005^g^	31.96 ± 0.057^no^	0.10 ± 0.00^q^
CSJ+	90	4.95 ± 0.010^oq^	11.92 ± 0.03^g‐i^	0.34 ± 0.005^g^	30.30 ± 0.264^q^	0.12 ± 0.001^op^
CSJ+	120	4.89 ± 0.010^q‐s^	11.86 ± 0.06^i‐j^	0.38 ± 0.010^uv^	29.10 ± 0.100^r^	0.17 ± 0.002^no^
CSJ0	0	5.24 ± 0.10^b‐g^	12.30 ± 0.00^ef^	0.30 ± 0.010^c^	36.20 ± 0.100^i^	0.08 ± 0.001^u^
CSJ0	30	5.22 ± 0.025^b‐f^	12.50 ± 0.00^e^	0.39 ± 0.011^tu^	34.33 ± 0.152^l^	0.10 ± 0.00^r^
CSJ0	60	5.11 ± 0.025^hk^	12.87 ± 0.06^cd^	0.71 ± 0.010^p^	33.10 ± 0.100^m^	0.12 ± 0.002^n^
CSJ0	90	5.02 ± 0.020^mn^	12.87 ± 0.10^c^	1.10 ± 0.015^k^	31.46 ± 0.057^op^	0.15 ± 0.002^l^
CSJ0	120	4.92 ± 0.020^p‐r^	13.56 ± 0.06^ab^	1.39 ± 0.011^g^	30.10 ± 0.100^q^	0.18 ± 0.002^h^
T1	0	5.30 ± 0.010^a^	12.50 ± 0.100^e^	0.35 ± 0.005^v^	38.80 ± 0.100^de^	0.11 ± 0.01^t^
T1	30	5.23 ± 0.010^a‐f^	12.20 ± 0.100^fg^	0.39 ± 0.011^tu^	36.03 ± 0.152^i^	0.12 ± 0.001^op^
T1	60	5.16 ± 0.010^f‐i^	11.90 ± 0.100^hi^	0.71 ± 0.010^p^	31.30 ± 0.100^f^	0.14 ± 0.001^m^
T1	90	5.09 ± 0.010^im^	11.60 ± 0.100^j^	1.10 ± 0.015^k^	26.26 ± 0.208^s^	0.17 ± 0.002^j^
T1	120	5.02 ± 0.01^m‐o^	11.30 ± 0.100^k^	1.39 ± 0.011^g^	20.40 ± 0.100^u^	0.20 ± 0.002^f^
T2	0	5.27 ± 0.005^ab^	12.80 ± 0.100^d^	0.40 ± 0.005^tu^	39.83 ± 0.288^c^	0.10 ± 0.00^r^
T2	30	5.25 ± 0.00^a‐d^	12.50 ± 0.100^e^	0.60 ± 0.015^q^	36.46 ± 0.057^hi^	0.13 ± 0.001^n^
T2	60	5.21 ± 0.005^b‐f^	12.20 ± 0.100^fg^	1.05 ± 0.010^l^	35.03 ± 0.057^jk^	0.16 ± 0.001^k^
T2	90	5.19 ± 0.005^c‐g^	11.90 ± 0.100^hi^	1.30 ± 0.005^i^	33.33 ± 0.152^m^	0.19 ± 0.001^g^
T2	120	5.18 ± 0.010^dh^	11.60 ± 0.100^j^	1.54 ± 0.005^lm^	32.13 ± 0.152^n^	0.22 ± 0.01^d^
T3	0	5.26 ± 0.010^a‐c^	13.10 ± 0.100^c^	0.41 ± 0.015^t^	40.06 ± 0.404^bc^	0.12 ± 0.001^op^
T3	30	5.17 ± 0.010^e‐h^	12.50 ± 0.100^e^	0.89 ± 0.005^u^	39.20 ± 0.100^d^	0.14 ± 0.002^m^
T3	60	5.08 ± 0.010^jm^	12.50 ± 0.100^e^	1.23 ± 0.010^fg^	38.60 ± 0.100^e^	0.16 ± 0.001^kl^
T3	90	4.99 ± 0.010^np^	12.20 ± 0.100^fg^	1.50 ± 0.005^ef^	37.83 ± 0.152^f^	0.17 ± 0.001^i^
T3	120	4.90 ± 0.010^qrs^	11.86 ± 0.057^ij^	1.76 ± 0.005^c^	37.16 ± 0.208^g^	0.19 ± 0.001^g^
T4	0	5.24 ± 0.010^a‐e^	13.40 ± 0.100^b^	0.46 ± 0.010^rs^	40.60 ± 0.173^ab^	0.14 ± 0.001^m^
T4	30	5.14 ± 0.010^g‐j^	13.10 ± 0.100^c^	0.93 ± 0.00^n^	38.50 ± 0.100^e^	0.16 ± 0.001^j^
T4	60	5.04 ± 0.010^l‐n^	12.80 ± 0.100^d^	1.29 ± 0.010^hi^	37 ± 0.100^gh^	0.19 ± 0.001^fg^
T4	90	4.94 ± 0.010^p‐r^	12.50 ± 0.100^e^	1.57 ± 0.00^e^	35.40 ± 0.100^h^	0.22 ± 0.001^d^
T4	120	4.84 ± 0.010^s^	12.16 ± 0.06^f‐h^	1.85 ± 0.010^b^	34.00 ± 0.100^l^	0.25 ± 0.001^c^
T5	0	5.22 ± 0.010^b‐f^	13.70 ± 0.100^a^	0.51 ± 0.010^r^	40.96 ± 0.057^a^	0.16 ± 0.00^k^
T5	30	5.11 ± 0.010^h‐l^	13.40 ± 0.100^b^	0.99 ± 0.010^m^	34.10 ± 0.100^l^	0.20 ± 0.001^e^
T5	60	4.99 ± 0.010^np^	13.10 ± 0.100^c^	1.34 ± 0.005^h^	29.13 ± 0.208^r^	0.24 ± 0.001^c^
T5	90	4.87 ± 0.010^rs^	12.80 ± 0.100^d^	1.65 ± 0.010^d^	23.10 ± 0.100^t^	0.29 ± 0.002^b^
T5	120	4.75 ± 0.010^t^	12.50 ± 0.100^e^	1.90 ± 0.005^a^	16.66 ± 0.152^v^	0.33 ± 0001^a^

*Note:* Means having similar letters in a column are statistically non‐significant, while having dissimilar letters are significant (*p* < 0.05).

Abbreviations: CSJ+, positive control; CSJ0, negative control; T1, 2 min Ultra‐sonication; T2, 4 min Ultra‐sonication; T3, 6 min Ultra‐sonication; T4, 8 min Ultra‐sonication; T5, 10 min Ultra‐sonication.

### 3.2 Ultrasonication Effect on Brix of Cantaloupe–Sugarcane Blended Juice

Table 2 demonstrates that ultrasonication significantly affected (*p* < 0.05) the brix of cantaloupe–sugarcane blended juice. Brix, or total soluble solids, is a crucial measure for the sensory characteristics of juices. Comparing sonicated and chemically preserved samples to the negative control revealed that the brix of the blended juice samples after sonication increased, however, chemically preserved samples had a decrease in the brix. The increase in juice brix may stem from several occurrences, including the release of intracellular compounds. The ultrasonication process can impair or disrupt intracellular constituents, such as sugars, acids, and other soluble compounds inside the juice (Zhang et al. 2024). Second, sonication may improve the extraction of soluble compounds. The disruption of the cell wall increases the extraction rate of soluble solids from the fruit's pulp and peel, leading to a higher brix value. Moreover, ultrasonication can partially evaporate water, leading to the concentration of soluble particles and an increase in brix value (Taha et al. 2024). Another factor that may enhance the brix value of juice is a method that inactivates enzymes responsible for the degradation of sugars and other soluble compounds. During ultrasonication, two primary enzymes, pectinases and amylases, become inactivated. The inhibition or inactivation of enzymes in juice maintains natural sugars and soluble solids, leading to an elevated brix value (Zou et al. 2010). It is seen that the brix level of blended juice declines after storage across all treatments, regardless of the preservation method, including the negative control. In conclusion, the sonication technique with duration of 5 min is an efficient method for preserving brix levels in juices. Nguyen and Nguyen (2018) maintained mulberry juice using sonication treatment for several durations and found that the brix levels after ultrasonication reached their peak, yielding results consistent with our observations. Bhat and Goh (2017) investigated the brix estimate of sonicated strawberry juice that was manually extracted. The study observed that sonication had no significant impact on brix, however the current research demonstrated a considerable alteration in brix when employing the ultrasonication technology for juice preservation. Gómez‐López et al. (2018) observed the impact of ultrasonication on passion fruit juices, finding that brix initially raises post‐sonication but then declines after storage; hence, this study validates our research.

### 3.3 Ultrasonication Effect on Vitamin C of Cantaloupe–Sugarcane Blended Juice

Sonication had significant effect on ascorbic acid (vitamin C) concentration in sugarcane and cantaloupe blended juice (Table 2). Vitamin C in different fruit juices can oxidize certain pathogens that may lead to human ailments. Ascorbic acid plays a crucial role in collagen formation, metal ion metabolism, immune system development, mitigation of cardiovascular disease and neurological disorders, and prevention of free radical‐induced DNA damage (Ali et al. 2024). At 0 day of storage vitamin C in CSJ0 was 36.20 mL/100 mL. When preservation techniques were applied then ascorbic acid increased except in positive control. Just after ultrasound treatment, highest vitamin C was observed in T5 (40.60 mL/100 mL) and lowest in CSJ+ (34.50 mL/100 mL). It is clear that ‘during storage’ vitamin C reduced in all treatments. After completion of storage period maximum retention of vitamin C was in T3 and minimum retention was in T5. After 120 days of storage vitamin C degraded abruptly in all treatments. It can be seen from the mean values of ascorbic acid that T3 (37.16 mL/100 mL) had highest concentration of ascorbic acid while T5 (16.66 mL/100 mL) has minimum concentration of ascorbic acid (Table 2). Different factors are responsible that increase vitamin C in blended juice by depleting dissolved oxygen through cavitations, without the application of heat. Compared to the control, sonicated samples preserved a greater amount of ascorbic acid. The preservation of ascorbic acid following sonication treatment is likely attributable to the elimination of dissolved oxygen necessary for the degradation of ascorbic acid during storage (Navida et al. 2022). Current study findings are supported by Navida et al. (2022) that during storage vitamin C decreased. According to results mentioned in the Table 2, vitamin C was more retained when sonication treatment was given for 6 min. It can be concluded that sonication for 6 min is suitable for maximum vitamin C retention during storage of sugarcane and cantaloupe blended juice. In investigations utilizing ultrasonography, it is shown that the ascorbic acid level in kiwi juice reduced, aligning with our current findings (Santhirasegaram et al. 2013). In a separate investigation, ascorbic acid levels rose following ultrasonic treatment of strawberry juice (Wang et al. 2019). It is possible because amino acids in the juice dissolves in the presence of gases (O_2_), whereas the ascorbic acid molecule interacts with free radicals and can be diminished by variables such as acoustic power and temperature impacts. Ultrasonication can increase vitamin C in juice up to some extent but when juices exposed for ultrasound it can cause degradation ascorbic acid (Merouani et al. 2015).

### 3.4 Effect of Sonication on the Titratable Acidity of Cantaloupe–Sugarcane Blended Juice

Titratable acidity quantifies the overall acidity of a solution, such as fruit juice. It is regarded as a significant issue in the beverage business, since it influences the sensory attributes and shelf stability of the product. The Table 2 demonstrates that ultrasonication significantly affected the acidity of blended juice (*p* < 0.05). The Table 2 illustrates the fluctuation in titratable acidity of pre‐ and post‐sonication treatment, as well as a comparison of sonicated and non‐sonicated treatments alongside positive and negative control samples. At day 0 of storage, both CSJ+ and CSJ0 exhibited an acidity of 0.30, but sonication resulted in a rise in acidity proportional to the duration of sonication. During the first storage period, the highest acidity was observed in treatment T5 (0.51), whereas the lowest was recorded in T1 (0.35). After 120 days of storage, the maximum acidity recorded was 1.90 in T5, while the lowest was 0.38 in CSJ+. The results indicated that chemical preservation, in contrast to sonication, provided superior control over the acidity levels in juice samples over the storage period. The acidity in the juice sample may be elevated for many causes. Sonication of juice causes the breakdown of cell walls in tissues, resulting in the release of several acidic chemicals, including tartaric acid, citric acid and malic acid (Taha et al. 2024). Sonication can enhance the extraction rate of acidic chemicals from pulp and elevate the sample's acidity. Enzymatic processes can be expedited during this procedure, resulting in elevated acidity levels (Zhang et al. 2024). Gómez‐López et al. (2018) examined the influence of ultrasonication on titratable acidity and found that acidity increased following sonication, corroborating our findings. Conversely, Abid et al. (2013) studied the impact of ultrasounds on several quality metrics of apple juice and found that it does not significantly affect the acidity, which might be due to the different parameters of sonication applied.

### 3.5 Ultrasonication Effect on Viscosity of Cantaloupe–Sugarcane Blended Juice

Viscosity quantifies a fluid's resistance to flow and is regarded as a significant aspect that affects the rheological qualities (texture, mouth feel, and overall quality) of the product. Table 2 indicates that sonication significantly (*p* < 0.05) affected the viscosity of sugarcane and cantaloupe blended juice. Table 2 further demonstrates that the viscosities of the blended juice samples increased with sonication duration. At 0 days of storage, the lowest viscosity was recorded in CSJ+ and CSJ0 (0.08 mPa.s), while Treatment T5 exhibited the highest viscosity (0.16 mPa.s). The results indicate that chemical preservatives first had little impact on viscosity, which subsequently began to rise progressively. Viscosity was shown to rise with prolonged sonication and storage duration. After 120 days of storage, T5 exhibited the highest viscosity of 0.24 mPa.s, whereas the negative control showed the lowest viscosity (0.13 mPa.s). The rise in viscosity can be due to ultrasonication treatment, which induces the gelation of pectin molecules, resulting in heightened viscosity. Pectin is a soluble fiber found in the cell walls of fruits. Sonication of the juice results in the formation of a gel‐like material and an increase in viscosity (Deng et al. 2024). Bosiljkov et al. (2009) indicate that viscosity increased with greater power application and prolonged sonication time. Furthermore, they found that an increase in power results in higher density rates of the soy‐based beverage, regardless of the treatment time. These results correspond with our findings. Chen et al. (2012) show that the viscosity of carrot juice sonicated at 58°C (1.63 mPa.s) displayed a statistically significant increase (*p* < 0.05), compared to the control sample (1.52 mPa.s). This result may be attributed to enhanced solubilization of pectin in cell walls, while these data also corroborate our study findings. It can be concluded that this study is in accordance with Chen et al. (2012) findings because in the current study viscosity also increased.

### 3.6 Effect of Sonication on the Total Phenolic Content of Cantaloupe–Sugarcane Blended Juice

Bioactive substances included in fruits and vegetables are highly advantageous for sustaining human health. Phenolic chemicals serve as antioxidants, advantageous for the human body, since they are preferentially oxidized over other cellular components and tissues, hence reducing cellular damage and aging (Alighourchi et al. 2013; Vella et al. 2019). Table 3 indicates that sonication processing noticeably affected the total phenolic content in cantaloupe–sugarcane blended juice (*p* < 0.05). Table 3 presents the influence of ultrasonography on several formulations, including comparisons to positive and negative controls. The overall phenolic content in blended juice rose rapidly after sonication processing. Chemical preservation (positive control) did not substantially influence total phenolic content in comparison to the negative control. On the first day of storage, the lowest total phenolic content was seen in CSJ0 (131.26 mg GAE/100 mL), while the highest was noted in T5 (170.83 mg GAE/100 mL). The results unequivocally demonstrate that an extension of sonication time is associated with an elevation in phenolic content, in the instance of T5 TPC content increased by 30.14% which is at maximum level. Subsequent to treatment exposure, juice samples were refrigerated at 4°Celsius for a period of 120 days. During the storage duration, total phenolic content significantly decreased across all treatments. The decrease in total phenolic content during storage may be ascribed to many mechanisms, including oxidation, enzymatic breakdown, and interactions with other compounds (Taha et al. 2024). Alighourchi et al. (2013) investigated the effect of sonication on the total phenolic content in pomegranate juice. The juice underwent sonication at various amplitudes (50%, 75%, and 100%) over time intervals of (0, 3, 6, and 9 min). Reports suggest that the total phenolic content increases with the use of sonication procedures in juices. Our results correspond with those reported by Alighourchi et al. (2013) subsequent to their research. This enhancement may arise from hydroxyl radicals generated by sonic cavitations, and dietary components, such as phenolic compounds, can undergo sonochemical hydroxylation (Zhang et al. 2024). The rising trends in phenolic content at particular amplitude levels and durations may be ascribed to the incorporation of hydroxyl radicals into the aromatic ring of phenolic compounds (Bhat et al. 2011). Phenolic chemicals are either associated with cellulose, hemicelluloses, and pectin, or they exist in a soluble state within the vacuole. The results clearly indicate that an increase in sonication duration correlates with a rise in the bioactive chemicals present in the juice. Del Socorro Cruz‐Cansino et al. (2015) investigated the potential of ultrasound to facilitate the release of these chemicals from the cell wall by inducing collapse by cavitations near colloidal particles, hence supporting our findings.

**TABLE 3:** The mean values±SD for the effect of treatments on the total phenolic content (mg GAE/100 mL) of blend (cantaloupe–sugarcane) juice during storage.

Treatments	Days				
	0	30	60	90	120
CSJ+	131.7 ± 0.153^c^	121.4 ± 0.569^t^	106.2 ± 0.361^fg^	84.20 ± 0.265^o^	57.36 ± 1.137^d^
CSJ0	131.3 ± 0.586^op^	110.1 ± 0.265^b^	85.46 ± 0.321^p^	63.76 ± 0.379^uv^	51.7 ± 0.611^gh^
T1	150.2 ± 0.100^h^	142.4 ± 0.265^f^	130.2 ± 0.0058^pq^	123.6 ± 0.529^s^	118.3 ± 0.577^u^
T2	156.7 ± 0.351^f^	149.8 ± 0.794^h^	140.2 ± 0.265^l^	134.4 ± 0.208^n^	126.3 ± 0.265^r^
T3	165.5 ± 0.252^c^	162.3 ± 0.231^d^	158.03 ± 0.153^ef^	154.6 ± 0.400^g^	149.1 ± 0.153^h^
T4	168.3 ± 0.173^b^	159.2 ± 0.289^e^	148.5 ± 0.265^ab^	141.1 ± 0.153^kl^	134.1 ± 0.115^n^
T5	170.8 ± 0.208^a^	155.4 ± 0.351^g^	142.2 ± 0.173^jk^	135.9 ± 0.306^m^	128.9 ± 0.208^q^

*Note:* Means having similar letters in a column or in a row are statistically non‐significant, while having dissimilar letters are significant (*p* < 0.05). Within box means express interaction means.

Abbreviations: CSJ+, positive control; CSJ0, negative control; T1, 2 min Ultra‐sonication; T2, 4 min Ultra‐sonication; T3, 6 min Ultra‐sonication; T4, 8 min Ultra‐sonication; T5, 10 min Ultra‐sonication.

### 3.7 Effect of Sonication on the Total Flavonoid Content of Cantaloupe–Sugarcane Blended Juice

Table 4 illustrates that sonication processing markedly affected the total flavonoid content in cantaloupe–sugarcane blended juice (*p* < 0.05). Flavonoids are naturally occurring polyphenolic substances present in plants, demonstrating a wide array of chemical and biological activities. Multiple studies have established a substantial correlation between elevated flavonoid intake and a reduced risk of cardiovascular disease and cancer (Liu et al. 2024). Sonication of the blended juice demonstrated that total flavonoid content increased with prolonged sonication duration. The mean measurements shown in Table 4 demonstrate that total flavonoid content increased following ultrasound therapy. Total flavonoid content began to diminish across all treatments during storage. Initially, the total flavonoid content was (51.33 mg CE/100 mL) in the negative control and (51.20 mg CE/100 mL) in the positive control, indicating a small decline in overall total flavonoid content. There was a notable alteration in total flavonoid contents with prolonged sonication duration. The total flavonoid content rose from 51.33 to 54.70 mg CE/100 mL following 5 min of sonication of the juice. This augmentation is facilitated by the technique of sonication. Fu et al. (2014) employed scanning electron microscopy to illustrate that ultrasonic treatment modified the surface architecture of plant material, hence aiding in the breakdown of plant cell walls. Cavitation produces hydroxyl and hydrogen free radicals by the dissociation of water molecules in aqueous solutions, triggered by the increased temperature and pressure resulting from the collapse of gas bubbles linked to cavitation. There are concerns over potential oxidative damage associated with free radicals, which is considered a disadvantage for the preservation of phenols; yet, it may enhance the antioxidant effectiveness of flavonoids (Taha et al. 2024). Guerrouj et al. (2016) indicates that sonication treatment of orange juice enhances flavonoid concentration. In comparison to the control sample (8.78 mg catechin equivalent/100 mL), all sonication treatments exhibited a considerable increase, ranging from 11.58 to 14.32 mg catechin equivalent/100 mL of juice. The research of Guerrouj et al. (2016) and the current study demonstrate that ultrasonic treatment can elevate the concentrations of bioactive compounds.

**TABLE 4:** The mean values±SD for the effect of treatments on the total flavonoid content (mg CE/100 mL) of blend (cantaloupe–sugarcane) juice during storage.

Treatments	Days				
	0	30	60	90	120
CSJ+	51.20 ± 0.200^no^	49.46 ± 0.378^r^	48.10 ± 0.00^u^	46.33 ± 0.057^v^	42.56 ± 0.115^s^
CSJ0	51.33 ± 0.115^no^	48.60 ± 0.264^t^	46.20 ± 0.100^v^	44.10 ± 0.100^fg^	39.50 ± 0.200^gh^
T1	52.06 ± 0.057^kl^	51.76 ± 0.057^lm^	50.20 ± 0.100^q^	49.06 ± 0.152^s^	48.36 ± 0.057^tu^
T2	52.83 ± 0.057^gh^	52.30 ± 0.100^k^	51.33 ± 0.057^no^	50.76 ± 0.057^p^	50.03 ± 0.115^q^
T3	53.70 ± 0.200^cd^	53.33 ± 0.057^de^	53.10 ± 0.00^e‐g^	52.86 ± 0.057^f‐h^	52.46 ± 0.057st
T4	54.20 ± 0.100^b^	53.23 ± 0.057^ef^	52.56 ± 0.057^h^	51.26 ± 0.057^no^	50.76 ± 0.057^p^
T5	54.70 ± 0.100^a^	53.93 ± 0.057^c^	52.66 ± 0.057^h^	51.43 ± 0.057^mn^	50.96 ± 0.057^op^

*Note:* Means having similar letters in a column or in a row are statistically non‐significant, while having dissimilar letters are significant (*p* < 0.05). Within box means express interaction means.

Abbreviations: CSJ+, positive control; CSJ0, negative control; T1, 2 min Ultra‐sonication; T2, 4 min Ultra‐sonication; T3, 6 min Ultra‐sonication; T4, 8 min Ultra‐sonication; T5, 10 min Ultra‐sonication.

### 3.8 Effect of Sonication on the DPPH Free Radical Scavenging Activity of Cantaloupe–Sugarcane Blend Juice

DPPH free radical scavenging activity assay is used to calculate the antioxidant activity of any substance. Table 5 indicates that ultrasound significantly influenced the DPPH free radical scavenging activity (%) of sugarcane and cantaloupe blended juice (*p* < 0.05). At day 0, CSJ0 exhibited a free radical scavenging activity of 74.36%. Upon applying sonication for varying durations (2, 4, 6, 8, and 10 min), it is evident that DPPH activity increased, with the peak value recorded in treatment T5 as 89.50%, while CSJ+ demonstrated the lowest DPPH activity. A notable (*p* < 0.05) improvement in DPPH free radical scavenging activity was seen in the T5 (89.50%) sample compared to both positive and negative controls. The increase in DPPH scavenging capacity in the juice samples was mostly attributed to the heightened concentrations of bioactive compounds during ultrasonication. Furthermore, the shear forces generated during ultrasound inactivated several oxidation‐related enzymes, enhancing the antioxidant capacity of the fluids. The rise following sonication is attributable to the augmented bioavailability of antioxidants through improved solubility and greater diffusion. When diffusion is augmented, it enables antioxidants to engage more efficiently with free radicals (Zhang et al. 2024). Ultrasonication can disrupt the cell wall and liberate bound antioxidants, including phenolic chemicals, flavonoids, and carotenoids (Taha et al. 2024). A similar study's results on DPPH free radical scavenging activity demonstrated that sonication improved the antioxidant efficacy of blended juices (Guerrouj et al. 2016). Present results are consistent with the findings of Ali et al. (2023) about the use of ultrasonography to the spinach juice. Oladunjoye et al. (2021) indicate that ultrasonication therapy significantly improves antioxidant activity levels with prolonged treatment time. The treatment with sonication increased the antioxidant activity of juice processed at 40°C, from 49.19% to 71.24%. The increase in antioxidant activity between 40°C and 50°C may be associated with the bioavailability of phenolic compounds during cavitation. A similar increase linked to the treatment condition has been seen in carrot and golden berry juice (Jabbar, Abid, Hu, Muhammad Hashim, et al. 2014; Nguyen and Nguyen 2018). Other authors have associated the increase in antioxidant activity with a decrease in the production of free hydroxyl radicals after the treatment of the juice component. Extended exposure to high concentrations of these radicals has been shown to negatively impact antioxidant activity (Wang et al. 2019; Yildiz and Feng 2019). This process may explain the decrease in antioxidant levels at 60°C. These results align with our findings. It is very important to note that treatment T3 had the highest DPPH (%) activity (77.06%) after 120 days of storage; it can be concluded that maximum antioxidant activity of juice blend can be achieved by optimization of ultrasonication time period.

**TABLE 5:** The mean values±SD for the effect of treatments on the DPPH‐free radicals scavenging activity (%) of blend (cantaloupe–sugarcane) juice during storage.

Treatments	Days				
	0	30	60	90	120
CSJ+	71.50 ± 0.500^j^	66.73 ± 0.642^m^	58.43 ± 0.513^q^	44.60 ± 0.556^r^	33.43 ± 0.450^s^
CSJ0	74.36 ± 0.321^h^	60.66 ± 0.288^p^	43.56 ± 0.404^r^	35.00 ± 1.00^s^	23.33 ± 0.763^t^
T1	74.83 ± 0.288^h^	72.16 ± 0.288^ij^	67.50 ± 0.500^klm^	64.43 ± 0.493^n^	62.33 ± 0.208^o^
T2	79.66 ± 0.288^ef^	77.16 ± 0.288^g^	71.50 ± 0.500^j^	68.40 ± 0.556^kl^	66.80 ± 0.100^lm^
T3	85.00 ± 1.00^b^	82.83 ± 0.288^c^	80.50 ± 0.500^de^	78.63 ± 0.450^fg^	77.06 ± 0.152^g^
T4	88.00 ± 0.00^a^	82.00 ± 0.500^cd^	77.73 ± 0.251^g^	73.46 ± 0.115^hi^	70.63 ± 0.208^j^
T5	89.50 ± 0.500^a^	82.00 ± 1.00^cd^	74.70 ± 0.556 ^h^	71.06 ± 0.665^j^	68.50 ± 0.100^k^

*Note:* Means having similar letters in a column or in a row are statistically non‐significant, while having dissimilar letters are significant (*p* < 0.05). Within box means express interaction means.

Abbreviations: CSJ+, positive control; CSJ0, negative control; T1, 2 min Ultra‐sonication; T2, 4 min Ultra‐sonication; T3, 6 min Ultra‐sonication; T4, 8 min Ultra‐sonication; T5, 10 min Ultra‐sonication.

### 3.9 Effect of Sonication on the Total Antioxidant Activity of Cantaloupe–Sugarcane Blend Juice

Table 6 demonstrates that sonication processing significantly influenced the total antioxidant activity in cantaloupe–sugarcane blended juice (*p* < 0.05). Following sonication of the blended juice, it was shown that antioxidant activity was significantly increased (Table 6). At the outset, the lowest total antioxidant activity was seen in treatment CSJ (102.50 mg TE/100 mL), whereas the highest was recorded in T3 (112.43 mg TE/100 mL). Total antioxidant activity rises with sonication duration, but only up to a certain threshold; beyond this limit, antioxidant activity began to decline. All juice samples were stored at refrigeration temperature, and it was noted that total antioxidant activity declined during storage. The impact of storage length on the total antioxidant levels in sugarcane and cantaloupe demonstrates that total antioxidants gradually decreased with extended storage time. Upon conclusion of the storage period, the maximum antioxidant activity was recorded in T3 (105.86 mg TE/100 mL), whereas the lowest was noted in the negative control (87.26 mg TE/100 mL). Various variables contribute to the increase in total antioxidant activity following sonication. Initially, sonication can activate enzymes that decompose cellular structures and liberate phenolic chemicals that exist in bound form. Second, ultrasonic waves can generate cavitation bubbles in juice that compromise the cell wall, releasing phenolic chemicals and thereby enhancing overall antioxidant activity. A significant discrepancy is apparent across treatments under storage circumstances (Taha et al. 2024; Zhang et al. 2024). Nadeem et al. (2018) found that all treatments demonstrated enhanced antioxidant activity in the carrot and grape juice mix compared to samples without ultrasonic treatment. The increased total antioxidant activity may be attributed to ultrasonic treatment. This method increased the concentrations of bound antioxidants, such as phenolic and ascorbic acid, leading to heightened antioxidant activity. Furthermore, ultrasonic treatment may inactivate enzymes, such as polyphenol oxidase, responsible for enzymatic browning, leading to increased total antioxidant activity levels. The results identified by these writers correspond with our present findings. Yildiz and Feng (2019) sought to investigate the effect of ultrasonic treatment on the physiological quality of cherry juice. The qualitative attributes of cherry juices, including total antioxidants, total phenolic content, and ascorbic acid concentrations, were analyzed comparatively. The results demonstrated that ultrasonic treatment significantly affected antioxidant activity, showing that the antioxidant capacity notably increased with prolonged sonication duration in cherry juice samples across all methods. These results also attest to our research findings.

**TABLE 6:** The mean values ± SD for the effect of treatments on the total antioxidant activity (mg TE/100 mL) of blend (cantaloupe–sugarcane) juice during storage.

Treatments	Days				
	0	30	60	90	120
CSJ+	104.3 ± 0.288^hi^	101.5 ± 0.305^kl^	94.70 ± 0.458^rs^	87.20 ± 0.100^c^	76.33 ± 0.208^o^
CSJ0	102.5 ± 0.500^c^	95.63 ± 0.416^q^	89.63 ± 0.450^v^	83.43 ± 0.305^de^	70.20 ± 0.655^l^
T1	106.5 ± 0.404^ef^	104.1 ± 0100^i^	101.23 ± 0.152^lm^	97.30 ± 0.100^f^	94.20 ± 0.00st
T2	108.6 ± 0.173^d^	105.1 ± 0.057^gh^	100.4 ± 0.100^mn^	95.16 ± 0.152^qr^	94.20 ± 0.100^u^
T3	112.4 ± 0.251^a^	111.1 ± 0.152^b^	101.2 ± 0.152^c^	108.10 ± 0.100^d^	105.8 ± 0.208^fg^
T4	110.1 ± 0.152^c^	107.2 ± 0.100^e^	102.2 ± 0.264^jk^	99.33 ± 0.152^o^	95.20 ± 0.100^qr^
T5	109.6 ± 0.100^c^	106.7 ± 0.100^ef^	99.56 ± 0.208^no^	93.40 ± 0.360^t^	87.26 ± 0.152st

*Note:* Means having similar letters in a column or in a row are statistically non‐significant, while having dissimilar letters are significant (*p* < 0.05). Within box means express interaction means.

Abbreviations: CSJ+, positive control; CSJ0, negative control; T1, 2 min Ultra‐sonication; T2, 4 min Ultra‐sonication; T3, 6 min Ultra‐sonication; T4, 8 min Ultra‐sonication; T5, 10 min Ultra‐sonication.

### 3.10 Effect of Sonication on the Total Plate Count of Cantaloupe–Sugarcane Blend Juice

The data in Table 7 illustrates the impact of treatment and storage duration on the total plate count of blended juice. The treatment and storage duration had a significant influence (*p* < 0.05) on the total plate count of blended juice. It can be seen from Table 7 that juice sample without any preservation technique had total plate count 2.70 ± 0.001 log_10_ CFU/mL while chemically preserved sample had bacterial count 2.38 ± 0.003 log_10_ CFU/mL. When ultrasonication treatment was applied, total plate count started to decrease as compared, positive and negative control. In the case of 2 min ultrasonication, the total plate count was noted as 2.20 ± 0.026 log_10_ CFU/mL and further reduced to 2.03 ± 0.016 log_10_ CFU/mL, when treatment time was extended up to 10 min. Microorganisms of juice sample increase in all treatments during storage of 120 days. It can be concluded that microorganisms were found to be reduced with the increase of ultrasonication time period. Among all treatments, T5 was most effective, to control microbes in blended juice sample to ensure safety of juice. Hashemi et al. (2024) performed ultrasonication (37 KHz, 300 W; 50°C, 60°C, and 70°C, 6 min) on celery juice to investigate the inactivation of *Escherichia coli* and *Salmonella typhi* . This research indicates that ultrasonication is highly successful in eradicating nearly all infections. Raising the temperature from 50°C to 70°C led to a 27.0% reduction in *Escherichia coli* and a 15.8% reduction in *Salmonella typhi* counts. Our results aligned with the findings of Shahid et al. (2025), who also noted a significant reduction in total plate count levels following the application of sonication to carrot juice. Likewise, Abid et al. (2014) noted a reduction in the total plate count values following the thermo‐sonication of apple juice. Their findings indicated that varying sonication frequencies and intensities are responsible for the physical breakdown of cell walls and the optimization of microbe elimination. All past finding and current research reporting proved that ultrasonication can be opted as a preservation technique to reduce microbial load in juices.

**TABLE 7:** The mean values ± SD for the effect of treatments on the total plate count (log10 CFU/mL) of blend (cantaloupe–sugarcane) juice during storage.

Treatments	Days				
	0	30	60	90	120
CSJ+	2.38 ± 0.003^lm^	2.48 ± 0.007^h‐j^	2.60 ± 0.012^f^	2.66 ± 0.010^e^	2.72 ± 0.008^d^
CSJ0	2.70 ± 0.001^de^	2.73 ± 0.004^d^	2.78 ± 0.007^c^	2.84 ± 0.001^b^	2.89 ± 0.004^a^
T1	2.20 ± 0.026^q‐s^	2.22 ± 0.039^q^	2.28 ± 0.011^o^	2.42 ± 0.001^kl^	2.51 ± 0.001^gh^
T2	2.17 ± 0.025^rs^	2.23 ± 0.018^pq^	2.27 ± 0.018^op^	2.45 ± 0.007^jk^	2.52 ± 0.001^gh^
T3	2.13 ± 0.016^tu^	2.20 ± 0.014^qr^	2.29 ± 0.007^o^	2.47 ± 0.003^ij^	2.53 ± 0.001^g^
T4	2.10 ± 0.020^u^	2.16 ± 0.012st	2.33 ± 0.003^n^	2.48 ± 0.002^h‐j^	2.54 ± 0.001^g^
T5	2.03 ± 0.016^v^	2.10 ± 0.013^u^	2.37 ± 0.005^mn^	2.50 ± 0.002^g‐i^	2.54 ± 0.009^g^

*Note:* Means having similar letters in a column or in a row are statistically non‐significant, while having dissimilar letters are significant (*p* < 0.05). Within box means express interaction means.

Abbreviations: CSJ0, negative control, CSJ+, positive control, T1, 2 min Ultra‐sonication; T2, 4 min Ultra‐sonication; T3, 6 min Ultra‐sonication; T4, 8 min Ultra‐sonication; T5, 10 min Ultra‐sonication.

### 3.11 Effect of Sonication on Yeast and Mold Count of Cantaloupe–Sugarcane Blend Juice

The mean values of the interaction among seven distinct treatments during 120 days of storage demonstrated significantly different outcomes for yeast and mold count of blend juice treatments (Table 8). The highest yeast and mold count was recorded in CSJ0 (2.60 ± 0.006 log_10_ CFU/mL) on the 0 day, while the lowest level was found in T1 (2.29 ± 0.004 log_10_ CFU/mL) on the 120 day of the research. The yeast and mold counts were much reduced in all samples except the untreated ones; however, in the ultrasound‐treated samples, these counts were well controlled due to the efficacy of sonication, as seen in Table 8. Yeast and mold counts were reduced after sonication as compared to other non‐sonicated treatments. Yeast and mold count was also reduced after sonication during storage of 120 days. A prior study found that using ultrasound in *aloe vera* juice might substitute the heat treatment procedure since sonication may effectively inactivate bacteria (Sasikumar 2015). The current findings were also associated with the results reported by Siddique et al. (2023), as they also observed lower yeast and mold counts in sonicated beverages. According to (Hussain, Batool, Yaqub, et al. 2024), sonication increases the antimicrobial compounds in the treated food products, hence increasing the shelf‐life of the products due to the reduced microbial loads.

**TABLE 8:** The mean values±SD for the effect of treatments on the yeast and mold count (log_10_ CFU/mL) of blend (cantaloupe–sugarcane) juice during storage.

Treatments	Days				
	0	30	60	90	120
CSJ+	2.32 ± 0.002^k^	2.45 ± 0.004^hi^	2.50 ± 0.008^ef^	2.60 ± 0.008^d^	2.66 ± 0.014^c^
CSJ0	2.60 ± 0.006^d^	2.69 ± 0.012^bc^	2.70 ± 0.008^b^	2.77 ± 0.004^a^	2.77 ± 0.004^a^
T1	2.09 ± 0.018^pq^	2.13 ± 0.009o	2.19 ± 0.011^n^	2.29 ± 0.004^kl^	2.42 ± 0.003^i^
T2	2.03 ± 0.008^r^	2.10 ± 0.010^op^	2.23 ± 0.006^m^	2.35 ± 0.003^j^	2.45 ± 0.001^hi^
T3	1.97 ± 0.018^s^	2.06 ± 0.007^qr^	2.26 ± 0.007^lm^	2.43 ± 0.003^i^	2.49 ± 0.001^fg^
T4	1.87 ± 0.029^u^	1.98 ± 0.013^s^	2.29 ± 0.004^kl^	2.45 ± 0.003^hi^	2.51 ± 0.002^ef^
T5	1.80 ± 0.021^v^	1.92 ± 0.010^t^	2.32 ± 0.002^k^	2.47 ± 0.002^gh^	2.52 ± 0.001^e^

*Note:* Means having similar letters in a column or in a row are statistically non‐significant, while having dissimilar letters are significant (*p* < 0.05). Within box means express interaction means.

Abbreviations: CSJ+, positive control; CSJ0, negative control; T1, 2 min Ultra‐sonication; T2, 4 min Ultra‐sonication; T3, 6 min Ultra‐sonication; T4, 8 min Ultra‐sonication; T5, 10 min Ultra‐sonication.

### 3.12 Effect of Sonication on the Sensory Evaluation of Cantaloupe–Sugarcane Blend Juice

**FIGURE 1:**
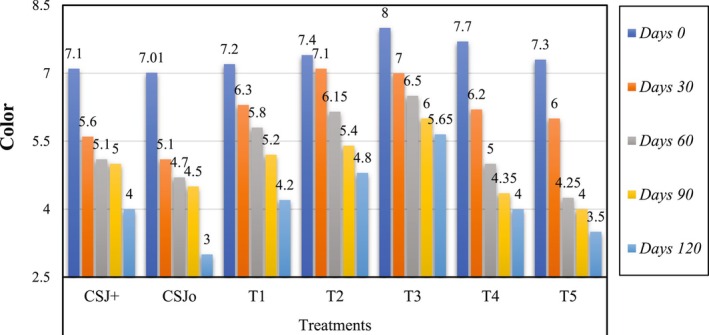
Effect of sonication treatments on the color of blend (cantaloupe–sugarcane) juice during storage.

**FIGURE 2:**
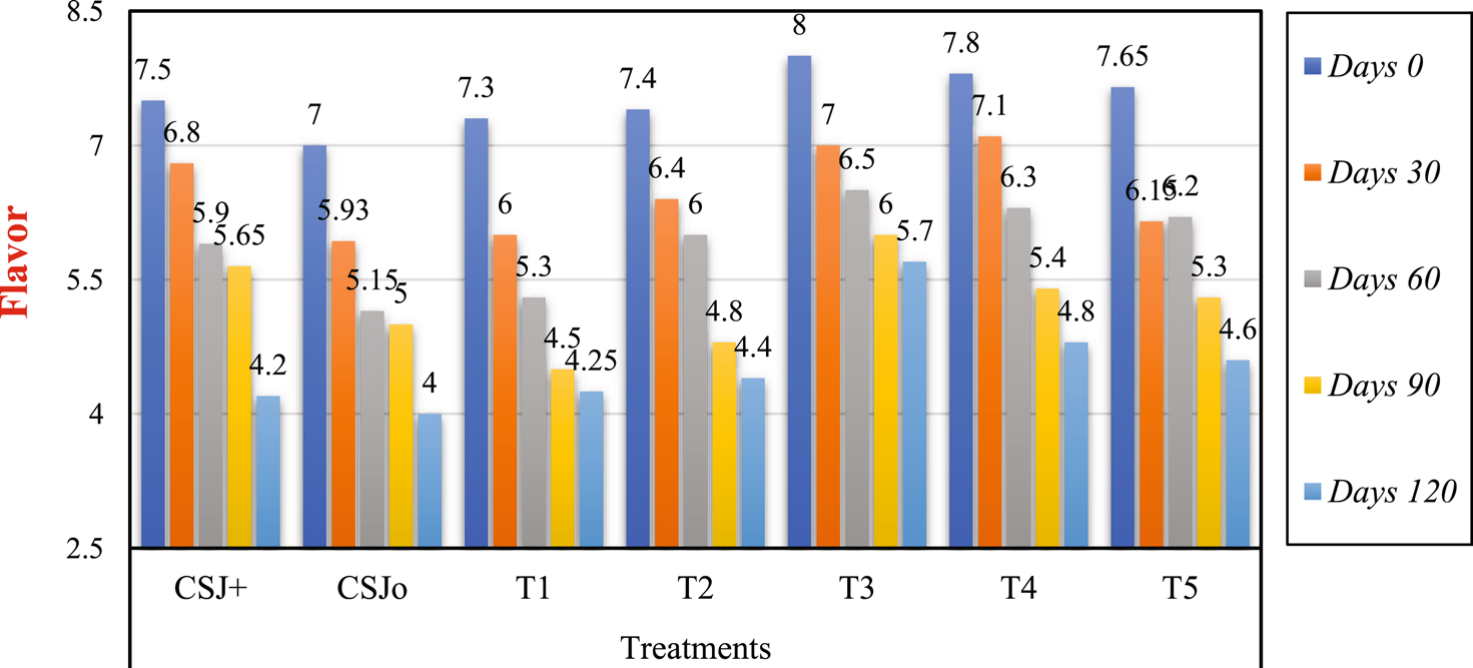
Effect of sonication treatments on the flavor of blend (cantaloupe–sugarcane) juice during storage.

**FIGURE 3:**
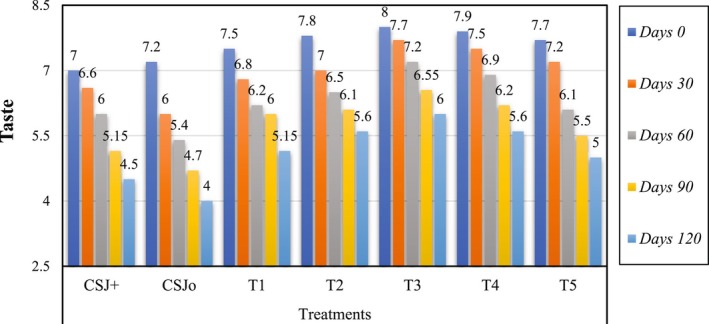
Effect of sonication treatments on the taste of blend (cantaloupe–sugarcane) juice during storage.

**FIGURE 4:**
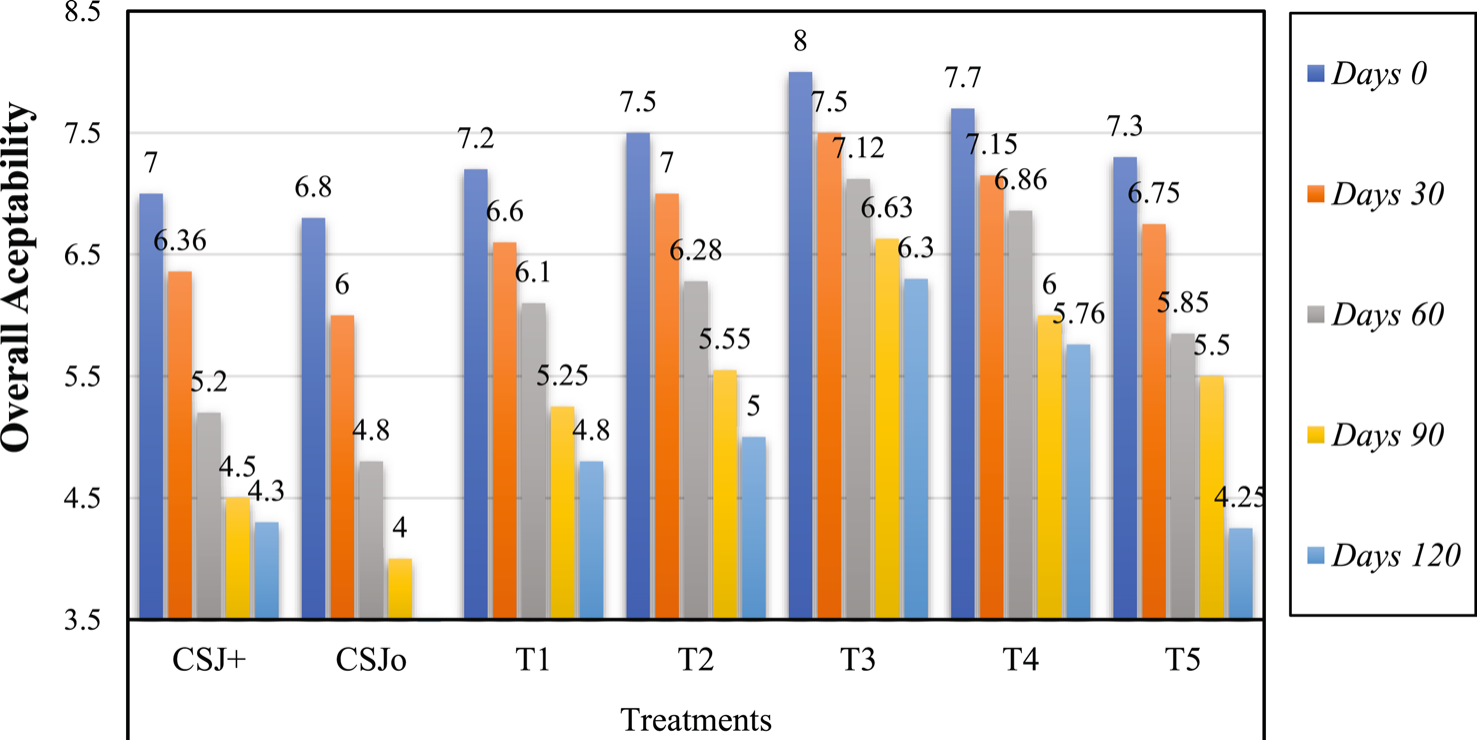
Effect of sonication treatments on the overall acceptability of blend (cantaloupe–sugarcane) juice during storage. CSJ0, Negative control; CSJ+, Positive control; T1, 2 min ultrasonication; T2, 4 min ultrasonication; T3, 6 min ultrasonication; T4, 8 min ultrasonication; T5, 10 min ultrasonication.

The sensory characteristics, including color, taste, flavor and overall acceptability, of all treatments were assessed, and the results are depicted in Figures 1, 2, 3, 4. Sensory evaluation of any final product is very important to ensure the quality and safety of the product. Sonicated juice samples attained higher scores by the panel with expertise in color, flavor, taste, and overall acceptability, when the sonication duration was extended to 6 min. A decline in the ratings of sensory parameters was observed when sonication was applied for 8 and 10 min. The acceptability of juice samples, in terms of all criteria, diminished with storage for both sonicated and non‐sonicated samples. It was seen that control juice sample was deteriorated more quickly as compared to the sonicated juice sample as well as chemically preserved sample. Following ultrasonication, T3 was identified as the superior treatment in terms of color, flavor, taste, and overall acceptability, with a score of 8. Similarly, analyzing and concluding the storage period scores of different treatments for 120 days, the T3 received the highest scores (5.65, 5.70, 6, and 6.3) for color, taste, flavor, and overall acceptability, respectively, whereas treatment CSJ0 attained the lowest scores (3, 4.25, 4, and 4) in the same categories. All assessed qualities parameters maintained the scores above the rejection threshold during the storage period at 4°C. Numerous investigations have demonstrated that sonication enhances the bitter flavor in citrus fruits. Ultrasonication considerably influences sensory ratings compared to pasteurized juices, as previously documented (Ahmad et al. 2023). The sensory evaluations of the thermosonicated samples in this investigation were shown to exceed those of the control and chemically preserved samples, and finding are in accordance of Ahmad et al. (2023) reporting. Reports on the impact of sonication on the sensory quality of fruit juices during storage are few, however several research have assessed its influence on instrumental color metrics. Sonication did not alter the color, flavor, or taste of sours of juice; although variations were seen in objective color parameters, they were imperceptible to the human eye (Dias et al. 2015). Similar results have also been reported by Gómez‐López et al. (2018), when sonication significantly affected the sensory parameters of passion fruit juice, as higher sonication times negatively affected the acceptability of the juice. Whereas, during the storage both sonicated and non‐sonicated juice samples received decreased sensory acceptance even during refrigerated storage. Our results are not supported by the findings of Dias et al. (2015), who reported non‐significant effect of sonication on the sensory scores of the juice, while in current research sensory properties are significantly affected by ultrasonication possibly due to high power and prolonged length of sonication.

## 4 Conclusion

This study examined the impact of different ultrasonication durations (while keeping power, frequency, and amplitude constant) on the physicochemical, phytochemical, microbial, and sensory characteristics of cantaloupe–sugarcane blend juice during 120 days of refrigerated storage. From the findings of the study, different ultrasonication durations significantly (*p* < 0.05) changed the physical, chemical, and phytochemical properties of the blended juice. Ultrasonication treatment improved the extraction of bioactive components, including total phenolic and total flavonoid content, from the cantaloupe–sugarcane blend. This treatment enhanced the juice's antioxidant activity, suggesting its potential health advantages. Moreover, ultrasonication findings represented that juice's physical characteristics, such as viscosity, density, and color were also changed. From findings of this study, the ultrasonication also affected the juice's chemical composition, resulting in notable alterations in its pH, titratable acidity, and total soluble solids concentration. From results of this technique, microbial load of the blend juice was significantly reduced, which even was observed lower during storage, as compared to the non‐sonicated juice samples. Ultrasonication for 6 min was proved to be optimum duration for good quality sensory results. This study underscores the efficacy of ultrasonication as a non‐thermal processing technique for improving the quality and nutritional content of fruit‐vegetable blended juices. The results of this research may facilitate the creation of innovative, value‐enhanced drinks with superior nutritional and sensory attributes. Subsequent research may concentrate on refining ultrasonication settings for certain fruit‐vegetable mixtures and examining the impact of ultrasonication on the microbiological safety and shelf life of these beverages.

## Author Contributions

**Abdul Rehman:** conceptualization (equal), formal analysis (equal), writing – review and editing (equal). **Muhammad Nadeem:** data curation (equal), formal analysis (equal). **Mian Anjum Murtaza:** supervision (equal), validation (equal), visualization (equal). **Safdar Hussain:** formal analysis (equal), investigation (equal), methodology (equal), supervision (equal). **Nida Firdous:** resources (equal), software (equal), writing – original draft (equal). **Rizwan Arshad:** investigation (equal), visualization (equal). **Ashiq Hussain:** supervision (equal), writing – original draft (equal), writing – review and editing (equal). **Abdeen Elsiddig Elkhedir:** project administration (equal), visualization (equal), writing – original draft (equal), writing – review and editing (equal).

## Ethics Statement

The sensory evaluation was conducted in accordance with the guidelines for sensory studies as outlined by the Ethics committee of Institute of Food Science and Human Nutrition, University of Sargodha, with the approval number (UOS/IFSN/2024/05). Informed consent was obtained from all individual participants included in the study.

## Consent

The authors have nothing to report.

## Conflicts of Interest

The authors declare no conflicts of interest.

## Data Availability

All data generated or analyzed during this study are included in this published article.
